# Complete Genome Sequence of a Duck Hepatitis A Virus 1 Isolated from a Pigeon in China

**DOI:** 10.1128/genomeA.00451-13

**Published:** 2013-07-11

**Authors:** Shaohua Shi, Hongmei Chen, Zhen Chen, Guanghua Fu, Chunhe Wan, Yu Huang, Su Lin, Longfei Cheng, Qiuling Fu, Jiansheng Lin, Fang Lin

**Affiliations:** Institute of Animal Husbandry and Veterinary Medicine, Fujian Academy of Agricultural Sciences, Fuzhou, Chinaa; Fujian Animal Diseases Control Technology Development Center, Fuzhou, Chinab

## Abstract

We report here the complete genome sequence of a duck hepatitis A virus 1 (DHAV-1), strain FJ1220, isolated from a dead pigeon in eastern China. DHAV-1 FJ1220 has high homology of up to 99.6% to the DHAV-1 strain Du/CH/LGD/111238 but relatively low homology to strains FFZ05 and FZ05. An amino acid hypervariable region in the VP1 protein of FJ1220 has the motif ^180^TPSGR^184^ replaced by ^180^ALSRG^184^ compared to strains FFZ05 and FZ05.

## GENOME ANNOUNCEMENT

Duck hepatitis (DH) is a highly fatal contagious infection in ducklings, characterized primarily by liver necrosis and hemorrhage, and it is caused mainly by three known types of duck hepatitis virus (DHV-1, DHV-2, and DHV-3) (1–3). DHV-1 is distributed worldwide, while DHV-2 and DHV-3 have been reported only in the United Kingdom and the United States, respectively (3). Recently, DHV-1 has been classified into a new genus, *Avihepatovirus*, of the family *Picornaviridae*, and designated duck hepatitis A virus 1 (DHAV-1). Two viruses associated by genotype and serotype with DHAV-1, now designated DHAV-2 and DHAV-3, have been identified separately in Taiwan and South Korea (4, 5). DHV-2 and DHV-3 have been classified into the family *Astroviridae* and designated duck astrovirus type I (DAstV-I) and DAstV-II, respectively (6).

In June 2012, the DHAV-1 strain FJ1220 was isolated from a pigeon farm that experienced a hepatitis outbreak with high mortality, up to 40%, in Fujian Province, eastern China. The nucleotide sequences of FJ1220 were amplified by reverse transcription-PCR (RT-PCR). Then the amplified products were purified and cloned into pMD18-T vectors (TaKaRa, Dalian, China) and sequenced with an ABI 3730 XL DNA analyzer. Sequences were assembled and analyzed by Lasergene v7.0 (DNASTAR, Inc.).

The full genomic length of DHAV-1 FJ1220 is 7,691 nucleotides (nt), excluding a 17-nt poly(A) tail at the 3′ end. A single large open reading frame (ORF), which is flanked by a 627-nt 5′ untranslated region (UTR) and a 315-nt 3′ UTR, is 6,750 nt in size. The single large ORF, which initiates from the optimal Kozak context at nt 626, encodes a putative polyprotein precursor of 2,249 amino acids. A total of 11 cleavage sites have been found in the polyprotein, contributing to generate 12 proteins (VP0, VP3, VP1, 2A1, 2A2, 2A3, 2B, 2C, 3A, 3B, 3C, and 3D) by the cleavage process. The 2A protein comprises three unrelated proteins, 2A1, 2A2, and 2A3. Among them, 2A1 has an NPG↓P cleavage site signal, 2A2 is an AIG1-like protein containing a GXXGXGKS nucleoside triphosphate (NTP)-binding motif, and 2A3, similar to the 2A proteins of parechoviruses, kobuviruses, and tremoviruses, has a special H-NC box.

Comparative genomic analysis showed that FJ1220 has high nucleotide similarity (99.6%) to DHAV-1 strain Du/CH/LGD/111238, which was collected in 2011. However, relatively low nucleotide homology (93.5%) occurs between FJ1220 and two strains, FFZ05 and FZ05, collected in the same place as FJ1220 in 2005. Amino acid sequence analysis revealed that a hypervariable region of DHAV-1 is located at amino acids (aa) 180 to 219 in capsid protein VP1, confirming the findings by Xu et al. (7). In the variable region of DHAV-1, the ^180^TPSGR^184^ motif of FFZ05 and FZ05 has been replaced by ^180^ALSRG^184^ in FJ1220. The genome data for strain FJ1220, isolated from a dead pigeon in eastern China and reported in our study, will be helpful for understanding the epidemiology and evolution of DHAV-1.

### Nucleotide sequence accession number.

The complete genome sequence of the DHAV-1 strain FJ1220 has been deposited in GenBank under the accession no. KC904272.
